# Association between gestational weight gain and perinatal outcomes among women with gestational diabetes mellitus

**DOI:** 10.3389/fendo.2025.1531814

**Published:** 2025-03-28

**Authors:** Zize Guo, Lihua Lin, Jiayi Dong, Juan Lin

**Affiliations:** ^1^ Department of Women’s Health Care, Fujian Maternity and Child Health Hospital, College of Clinical Medicine for Obstetrics & Gynecology and Pediatrics, Fujian Medical University, Fuzhou, Fujian, China; ^2^ Department of Healthcare, Fujian Maternity and Child Health Hospital, Fuzhou, Fujian, China; ^3^ Department of Women’s Health Care, Fujian Obstetrics and Gynecology Hospital, Fuzhou, Fujian, China; ^4^ Department of Obstetrics, Fujian Maternity and Child Health Hospital, Fuzhou, Fujian, China

**Keywords:** gestational diabetes mellitus, gestational weight gain, singleton pregnancy, adverse outcomes, body mass index

## Abstract

**Objective:**

This study identifies the optimal gestational weight gain (GWG) range for women with gestational diabetes mellitus (GDM) in singleton pregnancies and examines the relationship between GWG patterns and perinatal outcomes.

**Methods:**

We included 18,548 pregnant women diagnosed with GDM via a 75g glucose tolerance test at Fujian Maternal and Child Health Hospital from 2011 to 2022. Data on demographics, GWG, delivery details, and maternal and infant outcomes were collected. Subjects were divided into training and validation sets (7:3 ratio) and classified by pre-pregnancy BMI: underweight, normal weight, overweight, and obese. Logistic regression in the training set was conducted to determine optimal GWG for each group, and examined the relationship between adverse outcomes and the Institute of Medicine(IOM), Chinese nutrition society(CNS), and study-derived (AOR) standards in the validation set.

**Results:**

Among participants, 17.0% pregnant women gained insufficient GWG, 49.2% gained appropriate GWG, and 33.9% with excessive GWG. The optimal GWG for underweight, normal weight, overweight, and women with obesity were 12-14 kg, 8-14 kg, 6-10 kg, and 2-4 kg, respectively. Insufficient GWG in IOM and AOR standard increased composite adverse outcomes among underweight women. Normal weight: Insufficient GWG per CNS and AOR increased composite adverse outcomes; excessive GWG per all standards increased adverse outcomes. Insufficient GWG per all standards reduced the risk of small-for-gestational-age (SGA) infants, while excessive GWG increased the risk of large-for-gestational-age (LGA) infants, gestational hypertension, and cesarean section. Overweight: Excessive GWG per CNS and AOR increased composite adverse outcomes; excessive GWG per all standards increased the risk of cesarean delivery. Obese: Insufficient GWG per IOM and CNS increased composite adverse outcomes.

**Conclusion:**

GWG significantly influences adverse pregnancy outcomes. Compared to IOM guidelines, CNS recommendations and study-derived GWG ranges are more suitable for Chinese women with GDM in singleton pregnancies.

## Introduction

Gestational Diabetes Mellitus (GDM) is one of the most common pregnancy complication characterized by hyperglycemia, primarily due to insulin resistance and inadequate pancreatic β-cell secretion (1, 2). The incidence of GDM has risen due to lifestyle changes and increasing rates of overweight and obesity (3). GDM is associated with various adverse pregnancy outcomes for both mothers and newborns, including cesarean section, preterm birth (PTB), low Apgar scores, large for gestational age (LGA), small for gestational age (SGA), neonatal respiratory distress syndrome, neonatal jaundice, and neonatal intensive care unit (NICU) admission (4).

GDM management includes health education, nutritional therapy, exercise, blood glucose monitoring, and medication (5). Monitoring gestational weight gain (GWG) is crucial as it reflects maternal fat accumulation, body fluid increase, and fetal growth (6). Research indicates that excessive maternal weight gain in GDM patients is linked to higher LGA incidence. Zheng et al. found increased risks of LGA, macrosomia, and cesarean delivery in patients exceeding IOM weight gain limits, excessive weight gain also heightened risks of gestational hypertensive disorders, while insufficient gain increased SGA incidence (7). Barnes et al. noted that post-GDM diagnosis weight gain exacerbates insulin resistance, with every 2 kg increase raising insulin therapy needs by 1.3-fold and LGA risk by 1.4-fold (8).

In 2009, the IOM published recommendations for GWG ranges classified according to preconception body mass index (BMI) (9).These recommendations are based on the World Health Organization (WHO) BMI classification and primarily reference non-Asian populations, making their applicability to Chinese pregnant women questionable (10, 11). In 2021, the Chinese Nutrition Society (CNS) published the “Chinese Women’s Pregnancy Weight Monitoring and Evaluation Standards,” which use the BMI classification specific to the Chinese population and provide different recommended weight gain ranges compared to the IOM (12). Although both the IOM guidelines and the Chinese standards offer recommendations for weight gain during pregnancy, their proposed GWG and weight gain range (WGR) goals are primarily based on non-GDM populations. Therefore, the applicability of these recommendations to women with GDM requires validation through evidence-based medicine.

The purpose of this study was to investigate the appropriate range of weight gain during pregnancy for women with GDM in singleton pregnancies in southern China and to assess the relationship between GWG and WGR and pregnancy outcomes in women with GDM. The aim was to provide a scientific reference for healthcare institutions to monitor and manage the weight of patients with GDM.

### Study design and population

This study included women with regular perinatal care at Fujian Provincial Maternity and Child Health Hospital from 2011 to 2022, diagnosed with GDM at 24-28 weeks via oral glucose tolerance test(OGTT), and admitted for delivery. Data were obtained from electronic medical records, covering basic characteristics, pre-pregnancy height and weight, number of pregnancies and deliveries, weight at each perinatal care, maternal weight before delivery, delivery records, and hospitalization records. We excluded those with congenital anomalies, death, or unknown newborn information and with incomplete medical records.

**Figure 1:**
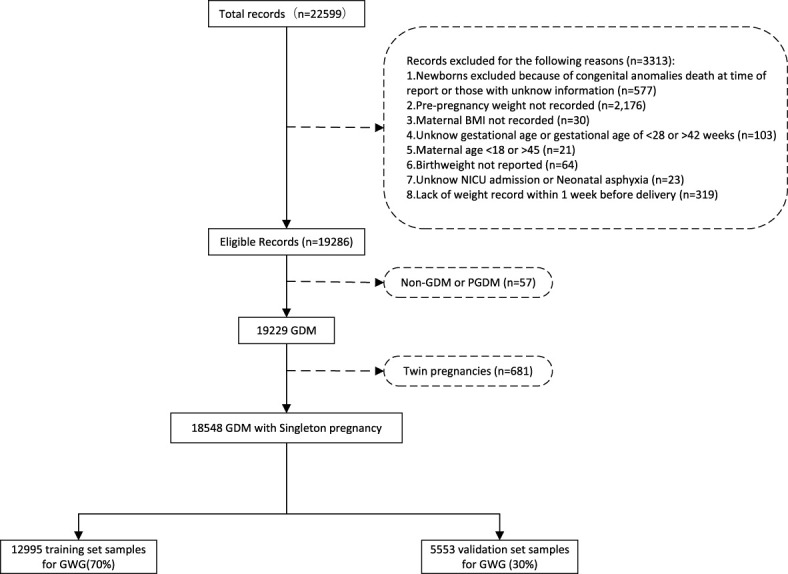
Flowchart of Subject selection.

A total of 22,599 pregnant women were initially retrieved. We excluded(**Figure 1**): congenital anomalies or neonatal death (n=577); unrecorded pre-pregnancy weights (n=2,176); missing pre-pregnancy BMI (n=30); deliveries with gestational age <28 or >42 weeks (n=103); maternal age <18 or >45 years (n=21); unknown birth weight (n=64); unknown post-delivery destination (n=23); missing weight records within 1 week before delivery (n=319); pre-pregnancy diabetes (n=57); and twin pregnancies (n=681). Finally, 18,548 pregnant women were included, randomized into a training set (n=12,995) and a validation set (n=5,553).

### Definition

Pre-pregnancy Body Mass Index (BMI) was calculated as [pre-pregnancy weight(kg)/height^2^(m^2^)] based onself-reported pre-pregnancy height and weight. Pre-pregnancy BMI was classified as underweight(BMI<18.5 kg/m²), normal weight(18.5≤BMI<23.9 kg/m²),overweight: (23.9≤BMI<27.9 kg/m²) andobesity(≥28.0 kg/m²) (13). According to relevant literature, women with GDM who have well-controlled glucose and no other complications are typically managed until full term (14, 15). For those with medication-controlled GDM, delivery is recommended between 39 0/7 and 39 6/7 weeks of gestation (16). Conversely, women with poorly controlled glucose should undergo earlier delivery (14, 15). However, current recommendations on the timing of delivery lack specific guidance (17). In light of this, the decision regarding the timing of delivery should balance the risks of preterm birth against the risk of stillbirth. Under these circumstances, delivery between 37 0/7 and 38 6/7 weeks of gestation may be reasonable (16). So we standardized the gestational weight gain (sGWG) for GDM equivalent to 37 weeks of gestational age, calculated by subtracting the pre-pregnancy weight from the weight in the week before delivery, dividing it by the actual number of weeks of gestation, and multiplying the result by 37 to estimate weight gain at 37 weeks of gestation (18). According to the criteria of monitoring and evaluation of pregnancy weight in Chinese women, the patterns of total weight gain in pregnant women were classified as adequate pregnancy weight gain (aGWG), inadequate pregnancy weight gain and excessive pregnancy weight gain (eGWG). GDM was diagnosed of GDM follows the criteria of OGTT proposed by the Association of Diabetes and Pregnancy Study Groups (IADPSG): fasting blood glucose ≥5.1 mmol/L, blood glucose ≥10.0 mmol/L after 1 hour, or blood glucose ≥8.5 mmol/L after 2 hours (19, 20). Newborns with a birth weight larger than the 90^th^ percentile or less than the 10^th^percentile were defined as LGA or SGA (21, 22). PTB was defined as delivery between 28 and 37 weeks of gestation (23). The gestational hypertensive disorders (GHDs) defined as blood pressure ≥140/90 mmHg that occurred after 20 weeks gestation but without proteinuria (24). A composite adverse outcome was defined as defined as either one of the above.

### Statistical analyses

Categorical variables are expressed as frequencies (percentages) and analyzed using the Chi-square test. Continuous variables are presented as mean ± standard or median (interquartile range), and analyzed using variance analysis. The absolute risk (AR) is calculated by dividing the number of adverse outcomes by the number of individuals in the specified range. In this study, ARs were calculated for the following: 1. AR under different standardized gestational weight gain (sGWG) conditions; 2. AR combining different sGWG and pre-pregnancy BMI.

In the training set, pregnant women were categorized into underweight, normal weight, overweight, and obese groups based on pre-pregnancy BMI. Within these four groups, sGWG was grouped by every 2.0 kg. Subsequently, the relationship between the respective weight gain ranges and composite adverse outcomes or specific adverse outcomes in each subgroup was evaluated, and the odds ratios (OR) and their 95% confidence intervals (CI) were calculated. A multivariable logistic regression model adjusted formaternal age, marital age, husband’s age, occupation, and conception method was used to explore the optimal range of gestational weight gain. The optimal gestational weight gain was defined as all weight gain categories showing a statistically significant protective association (OR < 1) with composite adverse outcomes. The appropriate GWG range identified in this study was defined as the AOR standard.

In the validation set, GWG among women with GDM was classified into three categories: insufficient, appropriate, and excessive, based on the ranges recommended by the IOM guidelines, CNS standards, and AOR standards. Using appropriate weight gain as the reference, OR and their 95% CI for insufficient and eGWG compared to appropriate weight gain in relation to adverse pregnancy outcomes were calculated to assess the association between aGWG and adverse outcomes under the three standards (IOM, CNS, AOR). When examining the relationship between weight gain patterns and specific adverse outcomes in different BMI groups, this study focused only on normal weight and overweight groups due to the small sample sizes in the underweight and obese groups. We explored the association between GWG and perinatal outcomes (LGA, SGA, PTB, GHDs, NICU admission, and cesarean delivery).

Statistical analyses and data visualization were performed using R software version 4.2.2, GraphPad Prism version 10.1.2, and Excel 2021. Two-sided tests were applied for all analyses, and differences were considered statistically significant at p-values <0.05.

## Results

### Basic characteristics of study population

This study included 18,548 singleton pregnant women with GDM. According to Chinese Nutrition Society(CNS)BMI definitions: 2,410 (13.0%) were underweight, 12,243 (66.0%) were normal weight, 3,142 (17.0%) were overweight, and 753 (4.0%) were obese. The average ages were (29.1 ± 4.3), (31.2 ± 4.6), (32.3 ± 4.6), and (31.8 ± 4.6) years, respectively. Gestational weeks at delivery were (38.5 ± 1.7), (38.5 ± 1.7), (38.3 ± 1.9), and (38.1 ± 2.1) weeks. Newborn birth weights were (3,141.6 ± 465.2) g, (3,252.8 ± 493.2) g, (3,308.7 ± 555.8) g, and (3,321.8 ± 613.0) g. Appropriate gestational weight gain proportions by CNS standards were 52.0%, 53.6%, 34.0%, and 30.8%, and by IOM standards were 44.4%, 38.7%, 38.5%, and 30.8%. All differences were statistically significant (P<0.001) (**Table 1**).

**Table 1:** Characteristics of selected participants.

	Underweight (N=2410)	Normal weight (N=12243)	Overweight (N=3142)	Obese (N=753)	*P*
Maternal age	29.1 ± 4.3	31.2 ± 4.6	32.3 ± 4.6	31.8 ± 4.6	<0.001
Occupation					
freelancer	1125 (46.7%)	5137 (42%)	1298 (41.3%)	352 (46.7%)	<0.001
Technical personnel	1032 (42.8%)	5830 (47.6%)	1515 (48.2%)	331 (44%)	
Administrative manager	99 (4.1%)	523 (4.3%)	108 (3.4%)	21 (2.8%)	
Business services employees	48 (2%)	291 (2.4%)	99 (3.2%)	18 (2.4%)	
Unknown	106 (4.4%)	462 (3.8%)	122 (3.9%)	31 (4.1%)	
Husband age	32.1 ± 3.4	33.1 ± 3.9	33.6 ± 4.1	33.6 ± 4.6	<0.001
Marriage age	26.1 ± 2.6	26.4 ± 2.8	26.5 ± 3.0	26.2 ± 3.1	<0.001
Pre-pregnancy weight	45.0 ± 3.3	53.9 ± 5.0	64.8 ± 5.0	77.2 ± 9.0	<0.001
Pre-pregnancy BMI	17.7 ± 5.3	21.1 ± 1.5	25.5 ± 1.1	31.3 ± 18.6	<0.001
Multipara					
No	1563 (64.9%)	6328 (51.7%)	1371 (43.6%)	349 (46.3%)	<0.001
Yes	847 (35.1%)	5915 (48.3%)	1771 (56.4%)	404 (53.7%)	
IOM gestational weight gain					
aGWG	1069 (44.4%)	4739 (38.7%)	1210 (38.5%)	232 (30.8%)	<0.001
iGWG	1075 (44.6%)	5232 (42.7%)	726 (23.1%)	171 (22.7%)	
eGWG	266 (11%)	2272 (18.6%)	1206 (38.4%)	350 (46.5%)	
CNSgestational weight gain					
aGWG	1254 (52%)	6568 (53.6%)	1067 (34%)	232 (30.8%)	<0.001
iGWG	648 (26.9%)	1599 (13.1%)	726 (23.1%)	171 (22.7%)	
eGWG	508 (21.1%)	4076 (33.3%)	1349 (42.9%)	350 (46.5%)	
Gestational age	38.5 ± 1.7	38.5 ± 1.7	38.3 ± 1.9	38.1 ± 2.1	<0.001
Mode of delivery					
Vaginal delivery	1814 (75.3%)	7696 (62.9%)	1607 (51.1%)	309 (41%)	<0.001
Cesarean section	596 (24.7%)	4547 (37.1%)	1535 (48.9%)	444 (59%)	
Birth weight	3141.6 ± 465.2	3252.8 ± 493.2	3308.7 ± 555.8	3321.8 ± 613.0	<0.001
SGA	117 (4.9%)	333 (2.7%)	79 (2.5%)	21 (2.8%)	<0.001
AGA	2244 (93.1%)	11348 (92.7%)	2805 (89.3%)	657 (87.3%)	
LGA	49 (2%)	562 (4.6%)	258 (8.2%)	75 (10%)	
Composite outcome					
No	2007 (83.3%)	9903 (80.9%)	2273 (72.3%)	446 (59.2%)	<0.001
Yes	403 (16.7%)	2340 (19.1%)	869 (27.7%)	307 (40.8%)	
PTB					
No	2198 (91.2%)	11159 (91.1%)	2799 (89.1%)	639 (84.9%)	<0.001
Yes	212 (8.8%)	1084 (8.9%)	343 (10.9%)	114 (15.1%)	
Macrosomia					
No	2361 (98%)	11681 (95.4%)	2884 (91.8%)	678 (90%)	<0.001
Yes	49 (2%)	562 (4.6%)	258 (8.2%)	75 (10%)	
Assisted reproductive					
No	2371 (98.4%)	11835 (96.7%)	3013 (95.9%)	734 (97.5%)	<0.001
Yes	39 (1.6%)	408 (3.3%)	129 (4.1%)	19 (2.5%)	
SGA					
No	2293 (95.1%)	11910 (97.3%)	3063 (97.5%)	732 (97.2%)	<0.001
Yes	117 (4.9%)	333 (2.7%)	79 (2.5%)	21 (2.8%)	
GHD					
No	2344 (97.3%)	11618 (94.9%)	2821 (89.8%)	585 (77.7%)	<0.001
Yes	66 (2.7%)	625 (5.1%)	321 (10.2%)	168 (22.3%)	

Date are presented as mean± standard deviation for continuous variables and n(%) for categorical variables.

BMI, body mass index; aGWG: appropriate gestational weight gain; iGWG:insufficient gestational weight gain; eGWG:excessive gestational weight gain; IOM: Institute of Medicine; PTB: preterm birth; LGA:large for gestational age; SGA: small for gestational age; GHD:gestational hypertensive disorders.

### The relationship between standardized gestational weight gain and adverse pregnancy outcomes

**Figure 2:**
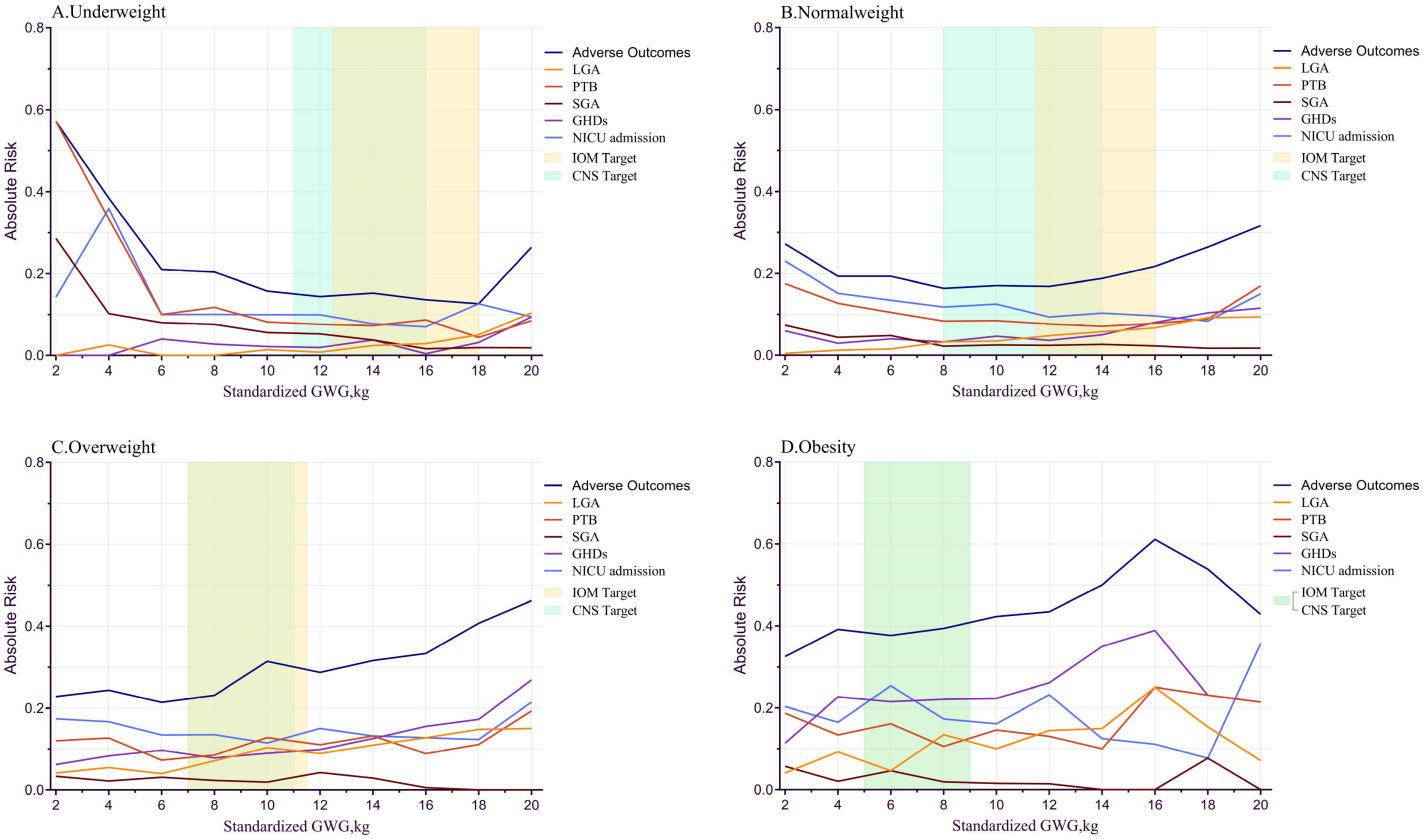
sGWG and absolute risk index for adverse outcomes in different pre-pregnancy body mass index. sGWG, standardized gestational weight gain; IOM, Institute of Medicine; PTB, preterm birth; LGA, large for gestational age; SGA, small for gestational age; GHDs, gestational hypertensive disorders; CNS, Chinese nutrition society; NICU, neonatal intensive care unit. **(A)** Underweight; **(B)** Normal weight; **(C)** Overweight; **(D)** Obesity.

**Figure 2** illustrates the relationship between GWG and the absolute risk (AR) of adverse outcomes. In the underweight group, AR increased significantly with weight gain <6 kg or >18 kg. For the normal weight group, AR showed a “U-shaped” relationship, increasing with weight gain <8 kg or >14 kg. In the overweight group, AR gradually increased with weight gain. In the obese group, AR decreased slightly with weight gain >16 kg, possibly due to the smaller sample size.

Exploring Specific Adverse Outcomes and sGWG: In the obese group, the curve oscillates when sGWG exceeds 16 kg, likely due to small sample size; hence, this part is not analyzed. LGA risk increases with sGWG in all groups. SGA risk significantly increases in the underweight group with sGWG <4 kg and decreases with increasing sGWG in other groups. PTB risk increases in the underweight group with sGWG <6 kg, showing a “U-shaped” distribution in normal and overweight groups. GHDs risk also shows a “U-shaped” distribution in underweight and normal weight groups but increases monotonically in the overweight group. NICU admission risk shows a “U-shaped” distribution in underweight and normal weight groups and an oscillatory curve in overweight and obese groups (**Figure 2**).

Comparison of IOM and CNS Recommended GWG Ranges: In the underweight group, IOM’s range is more lenient than CNS, with higher sGWG values. For the normal weight group, CNS’s lower GWG limit is lower than IOM’s, and the IOM standard shows a higher overall risk of adverse outcomes. In the overweight group, no significant difference exists between the standards, and the risk of composite adverse outcomes rises, indicating both IOM and CNS standards may not be applicable to the overweight GDM population (**Figure 2**).

**Figure 3:**
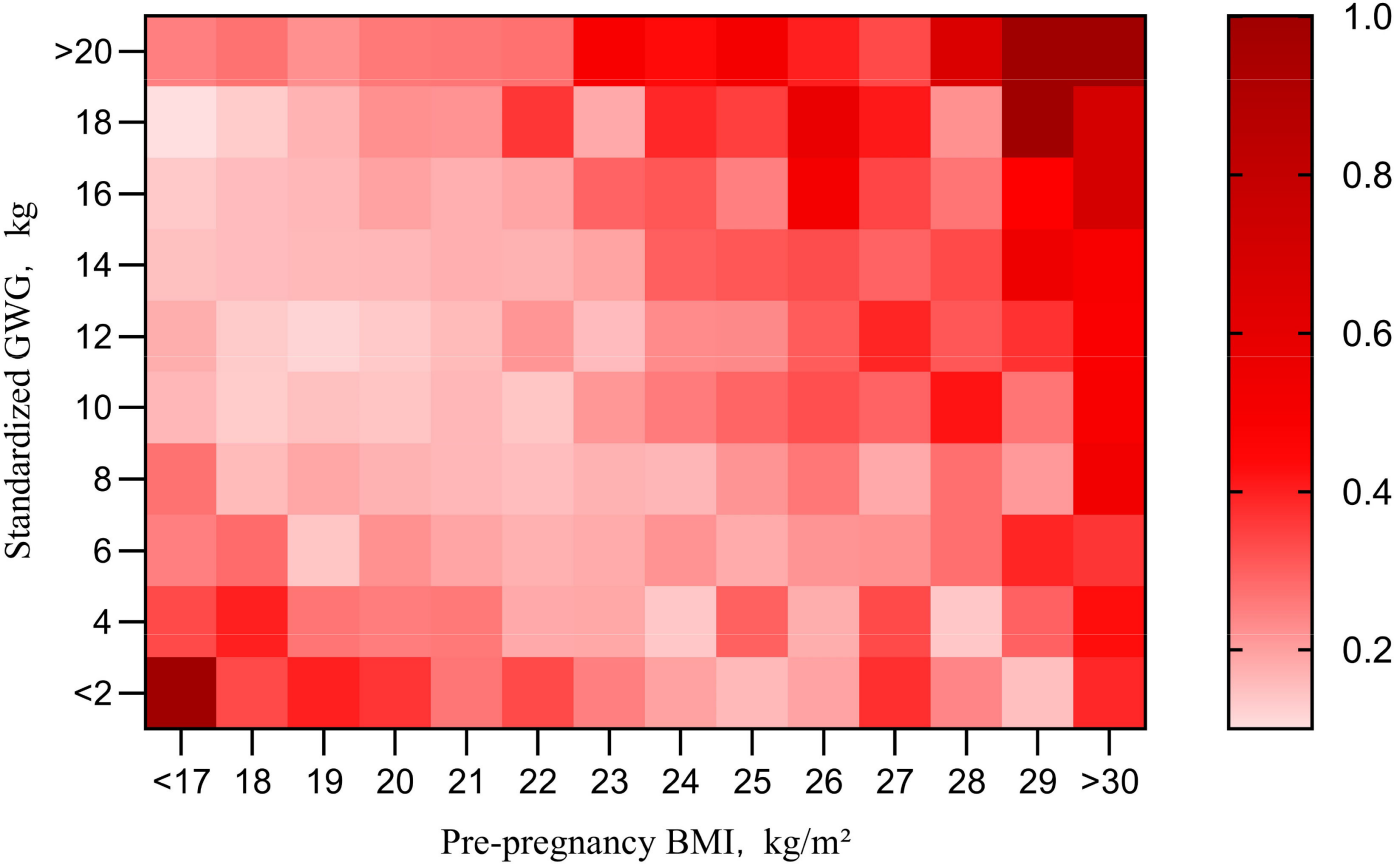
Pre-pregnancy BMI and sGWG absolute risk index for adverse outcomes. BMI, body mass index; GWG, gestational weight gain.

Dividing the sample into four groups by pre-pregnancy BMI shows the impact of gestational weight gain on adverse outcomes but overlooks the effect of BMI itself. To explore the combined impact of pre-pregnancy BMI and gestational weight gain, a heat map of AR was created (**Figure 3**). Each square represents AR within a specific range of pre-pregnancy BMI and sGWG. When pre-pregnancy BMI exceeds 28 kg/m² and sGWG exceeds 14 kg, the risk of adverse outcomes approaches 1. Similarly, when pre-pregnancy BMI is less than 18 kg/m² and sGWG is less than 6 kg, the risk also approaches 1 (**Figure 3**).

### Determination of the appropriate gestational weight gain range and its relationship with adverse pregnancy outcomes

**Figure 4:**
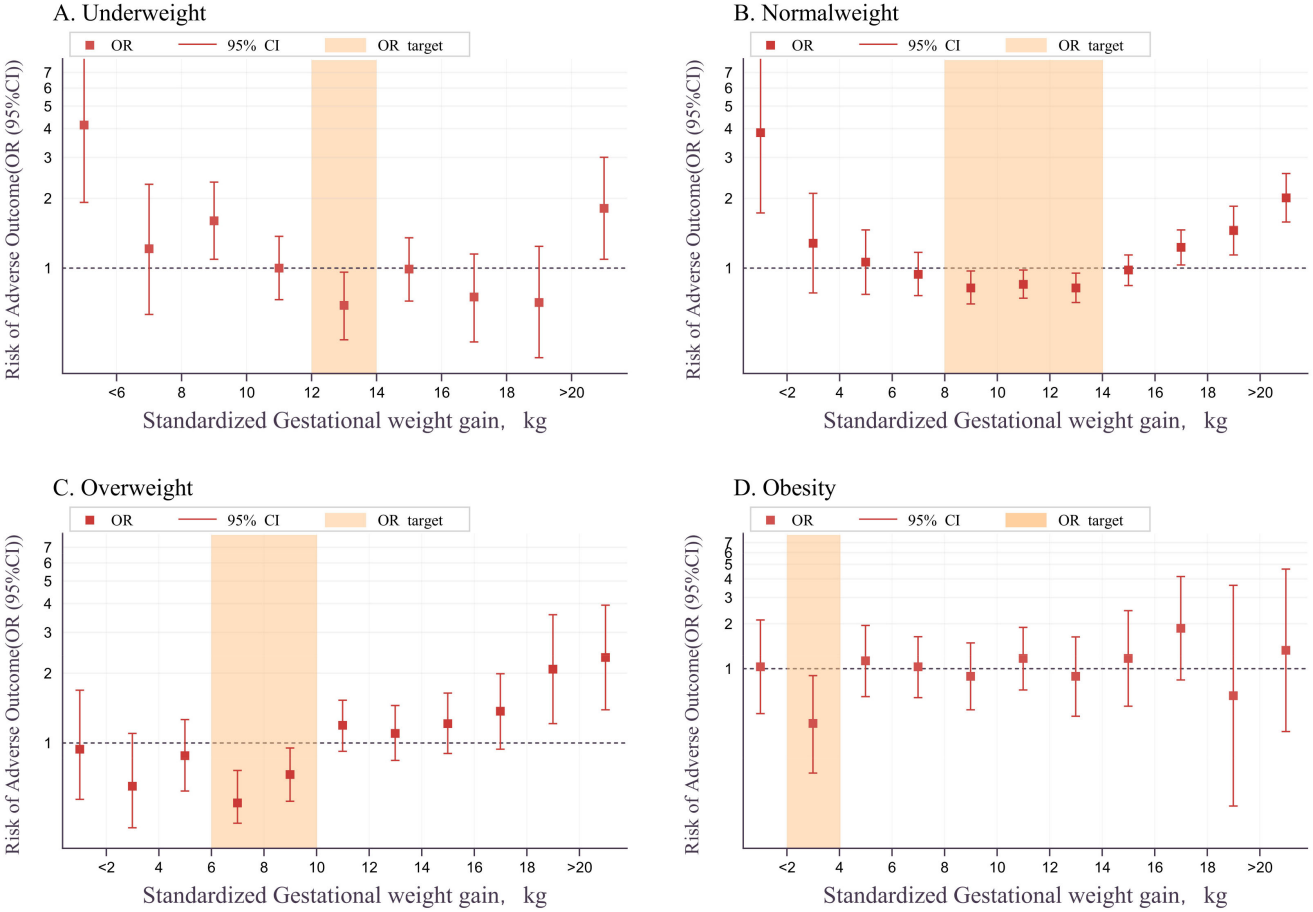
Relationship between sGWG and composite adverse outcomes in BMI categories. BMI, body mass index; sGWG, standardized gestational weight gain. **(A)** Underweight; **(B)** Normal weight; **(C)** Overweight; **(D)** Obesity.

A multivariable logistic regression model in the training set analyzed the relationship between sGWG and composite adverse outcomes. Results showed that sGWG of 12-14 kg for underweight, 8-14 kg for normal weight, 6-10 kg for overweight, and 2-4 kg for women with obesity significantly reduced the risk of composite adverse outcomes (**Table 2** and **Figure 4**).

**Table 2:** Association between sGWG and risk of composite outcomes in pregnant women with different preconception BMI.

	Underweight (n=1777)	Normal weight (n=8614)	Overweight (n=2106)	Obese (n=498)
sGWG	OR (95%CI)	OR (95%CI)	OR (95%CI)	OR (95%CI)
<2kg		3.84 (1.73-8.51)*	0.94 (0.52-1.69)	1.03 (0.50-2.12)
2-4kg		1.28 (0.78-2.10)	0.65 (0.38-1.10)	0.43 (0.20-0.90)*
4-6kg	4.15 (1.92-8.93)#*	1.06 (0.77-1.46)	0.88 (0.62-1.26)	1.13 (0.65-1.95)
6-8kg	1.21 (0.63-2.30)	0.94 (0.76-1.17)	0.55 (0.40-0.76)*	1.03 (0.64-1.64)
8-10kg	1.60 (1.09-2.35)*	0.82 (0.70-0.97)*	0.73 (0.56-0.95)*	0.89 (0.53-1.49)
10-12kg	1.00 (0.73-1.37)	0.85 (0.74-0.98)*	1.19 (0.92-1.53)	1.17 (0.72-1.89)
12-14kg	0.69 (0.49-0.96)*	0.82 (0.71-0.95)*	1.10 (0.84-1.45)	0.89 (0.48-1.63)
14-16kg	0.99 (0.72-1.35)	0.98 (0.84-1.14)	1.21 (0.90-1.64)	1.17 (0.56-2.45)
16-18kg	0.75 (0.48-1.15)	1.23 (1.03-1.46)*	1.37 (0.94-1.99)	1.86 (0.84-4.14)
18-20kg	0.71 (0.41-1.24)	1.45 (1.14-1.85)*	2.08 (1.21-3.58)*	0.66 (0.12-3.62)
>20kg	1.81 (1.09-3.01)*	2.01 (1.58-2.56)*	2.34 (1.39-3.94)*	1.33 (0.38-4.65)

#pregnant women with sGWG<6kg in underweight group were combined into one group; *p<0.05.

BMI, body mass index; sGWG, standard gestational weight gain; OR, odds ratio; CI, confdence interval; kg, kilogram.

We calculated the aGWG range for different BMI groups. For the underweight group, the recommended lower limit is slightly higher and the upper limit slightly lower than the CNS standard. The normal weight group’s range matches the CNS standard. For the overweight group, the range is slightly lower than CNS, and for the obese group, it is even lower (**Table 3**).

**Table 3:** The guidelines of gestational weight gain in different standard.

GWG guideline	Underweight (kg)	Normal weight (kg)	Overweight (kg)	Obese (kg)
IOMguideline	12.5-18	11.5-16	7-11.5	5-9
CNSguideline	11-16	8-14	7-11	5-9
AOR recommendation	12-14	8-14	6-10	2-4

GWG, gestational weight gain; IOM, Institute of Medicine; CNS, Chinese Nutrition Society; AOR, appropriate gestational weight gain range.

**Figure 5:**
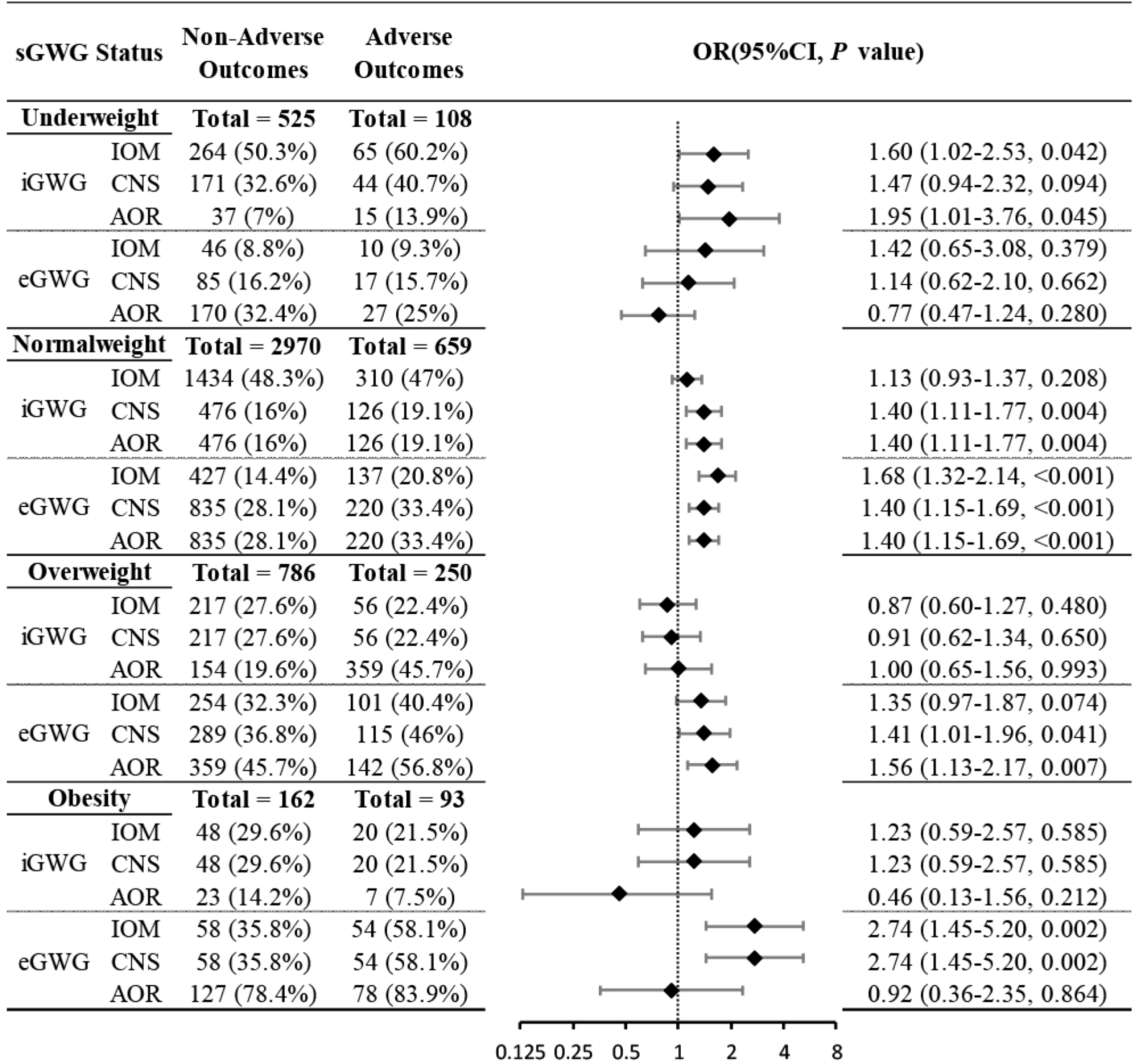
Association between sGWG patterns and composite adverse outcomes among pregnant women in different pre-pregnancy BMIs. BMI, body mass index; sGWG, standardized gestational weight gain.

The relationship between sGWG patterns and composite adverse outcomes was explored in the validation set under IOM, CNS, and AOR standards. In the underweight group, iGWG increased the risk of adverse outcomes under IOM and AOR standards (P < 0.05), but not CNS (P > 0.05). In the normal weight group, iGWG increased risk under CNS and AOR, and eGWG increased risk under all three standards (P < 0.05). In the overweight group, iGWG did not increase risk under any standard (P > 0.05), but eGWG increased risk under CNS and AOR (P < 0.05). In the obese group, iGWG showed no association with adverse outcomes, but eGWG increased risk under IOM and CNS standards. Thus, in overweight and obese groups, insufficient weight gain did not increase risk, but excessive weight gain did (**Figure 5**).

**Figure 6:**
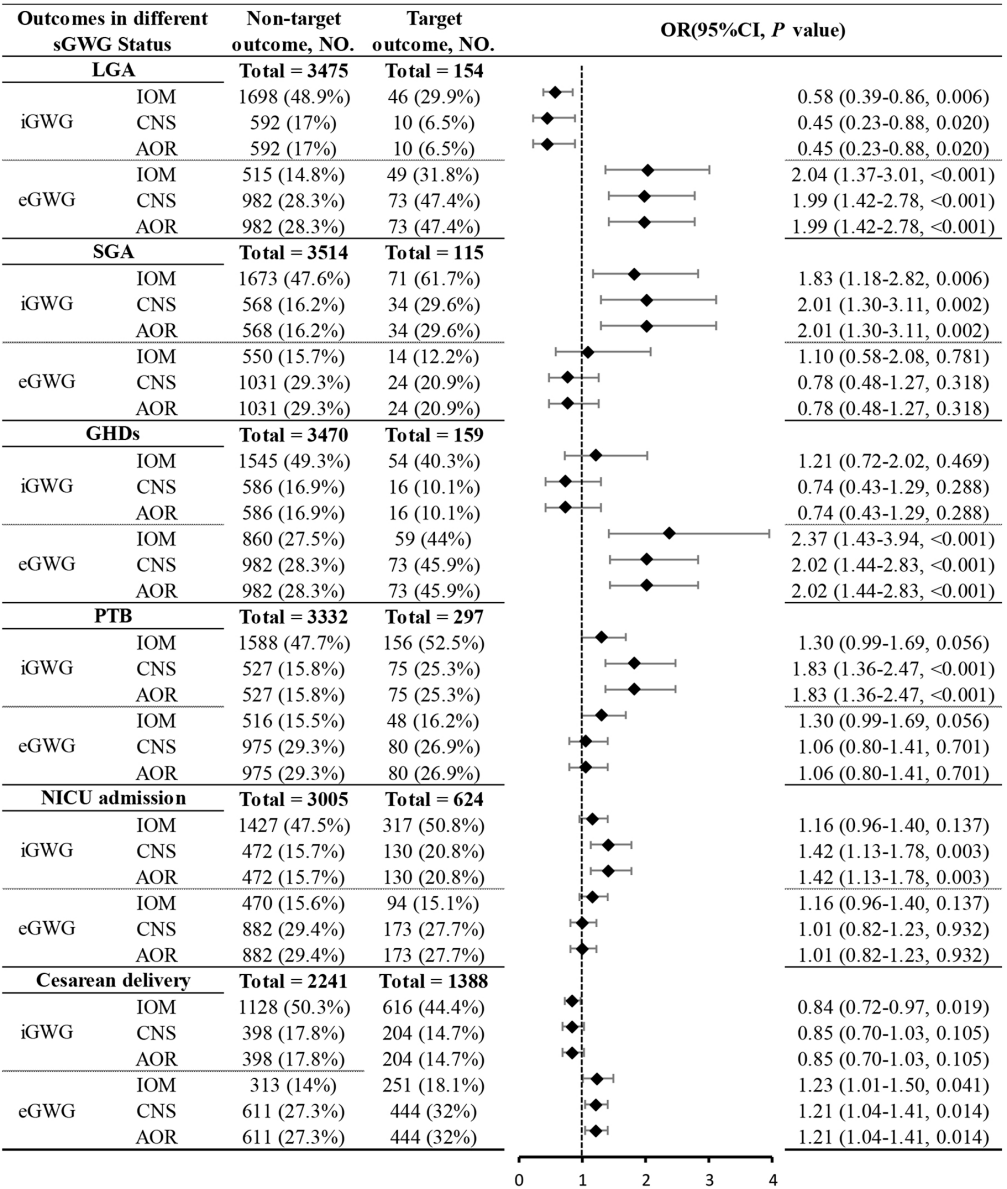
Association between sGWG patterns and composite adverse outcomes among pregnant women with normal weight. sGWG, standardized gestational weight gain.

**Figure 7:**
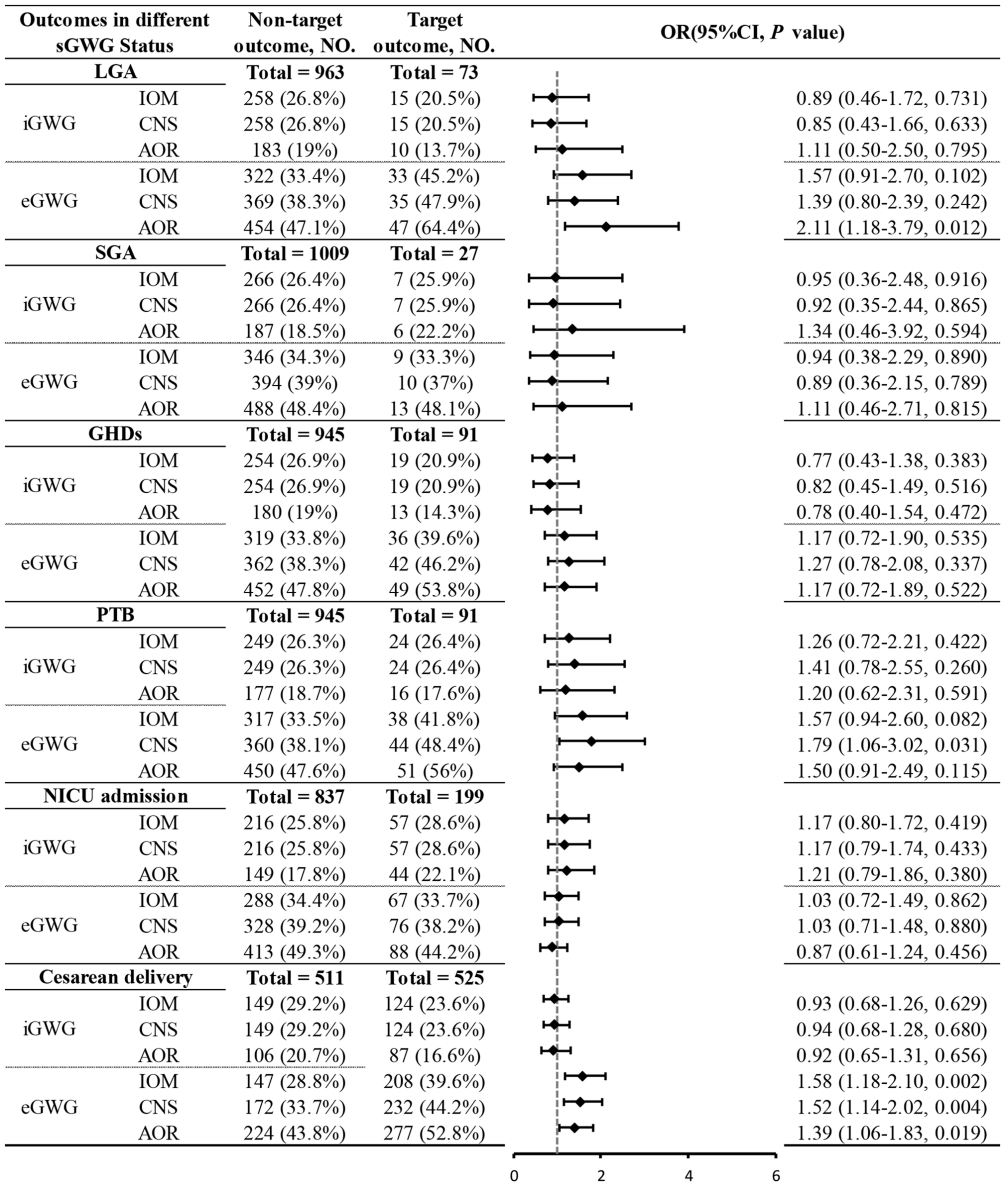
Association between sGWG patterns and composite adverse outcomes among pregnant women with overweight. sGWG, standardized gestational weight gain.

Due to sample size limitations in the underweight and obese groups, the relationship between sGWG and specific adverse outcomes was only analyzed in the normal weight and overweight groups. Normal Weight Group: iGWG under IOM, CNS, and AOR standards increased the risk of SGA and decreased the risk of LGA (P < 0.05). No significant association was found between iGWG and GHDs (P > 0.05), but eGWG increased GHDs risk (P < 0.05). iGWG under CNS and AOR standards increased the risk of PTB and NICU admission (P < 0.05). iGWG under IOM standard was associated with decreased cesarean section risk (P < 0.05); eGWG increased cesarean risk under CNS and AOR standards (P < 0.05) (**Figure 6**).Overweight Group: iGWG under IOM, CNS, and AOR standards showed no significant differences in adverse outcomes like LGA, SGA, GHDs, PTB, NICU admission, and cesarean section (P > 0.05). eGWG increased cesarean section risk (P < 0.05) and LGA risk under AOR standard (P < 0.05). eGWG under CNS standard increased PTB risk (P < 0.05) (**Figure 7**).

## Discussion

Rising obesity rates among women of childbearing age increase the incidence of GDM, posing significant threats to maternal and neonatal health (20, 25). Besides blood glucose levels, pre-pregnancy BMI and eGWG are independent risk factors for pregnancy complications (26). Both GWG and hyperglycemia are key modifiable factors that contribute to adverse outcomes of GDM during pregnancy (27). High pre-pregnancy BMI and eGWG are closely linked to negative health outcomes, making healthy GWG a critical topic in international public health (28–30). In 2009, the IOM) recommended the following healthy GWG ranges based on pre-pregnancy BMI: 12.5-18 kg for underweight (BMI < 18.5 kg/m²), 11.5-16 kg for normal weight (BMI 18.5-24.9 kg/m²), 7-11.5 kg for overweight (BMI 25-29.9 kg/m²), and 5-9 kg for obese (BMI ≥ 30 kg/m²) (29). However, these may not suit Asian populations due to differences like shorter stature and higher body fat percentage (31, 32). In October 2021, the CNS released the “Weight Monitoring and Evaluation During Pregnancy for Chinese Women” standard, which offers GWG ranges based on the BMI classification for the Chinese population: 11-16 kg for underweight (BMI < 18.5 kg/m²), 8-14 kg for healthy weight (BMI 18.5-23.9 kg/m²), 7-11 kg for overweight (BMI 24-27.9 kg/m²), and 5-9 kg for obese (BMI ≥ 28 kg/m²) (12).In a large cohort study in China, Chen et al. included 3,170 term singleton pregnant women (gestation 37 to 42 weeks) and compared GWG status based on the 2009 IOM guidelines and the 2021 CNS guidelines. Results showed that, according to the CNS standards, the proportions of insufficient, appropriate, and excessive GWG were 14.1%, 48.1%, and 37.9%, respectively; whereas, according to the IOM guidelines, these proportions were 39.7%, 37.2%, and 23.1%, indicating that GWG classification varies with different standards, consistent with previous findings (33). The wide range of recommended GWG means that individuals at the borderline of BMI classifications might receive different GWG classifications despite similar risks of adverse outcomes. For example, per IOM standards, a woman with a BMI of 24.9 kg/m² and a total GWG of 12 kg would be classified as having aGWG, while a woman with a BMI of 25.1 kg/m² with the same GWG would be classified as having eGWG. This highlights the variability in study populations, statistical methods, results, and conclusions regarding the optimal range of GWG in previous research (34).

This study defines a more precise range of aGWG for singleton pregnancies with GDM across different BMI categories: 12-14 kg for the underweight group, 8-14 kg for the normal weight group, 6-10 kg for the overweight group, and 2-4 kg for the obese group. Based on data from 8,103 GDM pregnant women in a tertiary hospital, Fan et al. determined optimal GWG ranges for different BMI categories in China, suggesting 11-17.5 kg for underweight, 3.7-9.7 kg for normal weight, 0.6-4.8 kg for overweight, and 0.6-4.8 kg for women with obesity. Their proposed ranges are stricter than those suggested in this study (35).

Compared to IOM standards, the GWG ranges derived from this study demonstrate higher precision and stringency. While similar to the CNS standards in indicating adverse pregnancy outcomes, they particularly emphasize the association between excessive weight gain and an increased risk of LGA in overweight pregnant women. Overall, the CNS and AOR standards are more effective than the IOM standards in predicting the risk of adverse outcomes. Some studies suggest that adopting stricter GWG targets than the IOM standards may improve maternal and fetal health outcomes (27). Furthermore, GWG targets optimized through statistical methods have shown better effectiveness in predicting adverse outcomes compared to IOM targets (36). These findings align with the results of this study. However, a study from Australia found that, although stricter GWG targets compared to IOM standards led to more women achieving appropriate GWG, there were no significant differences in the incidence of insulin resistance, preterm birth, shoulder dystocia, cesarean section, LGA, macrosomia, SGA, neonatal hypoglycemia, neonatal jaundice, and abnormal maternal blood glucose levels postpartum (37). This may be due to the combined effect of pre-pregnancy BMI, GDM, and GWG on pregnancy outcomes (38). In the overweight pregnant women group, whether weight gain was above or below the recommended standards, it did not effectively predict the risk of adverse outcomes. This suggests that existing standards are not suitable for reducing the occurrence of related adverse risks in this particular group. The aforementioned studies are based on retrospective data, and a more rigorous prospective design is needed to propose appropriate weight gain ranges.

This study shows a correlation between GWG and outcomes like LGA, SGA, PTB, GHDs, and NICU admission across different pre-pregnancy BMI groups. In underweight and normal weight women, LGA risk increases with GWG. Studies indicate that for every 2 kg of weight gain above the recommended limit, LGA risk increases by 44%, even with intensive glucose management (8). Excessive weight gain in GDM leads to higher fasting glucose and insulin therapy rates, due to increased insulin resistance. Viecceli C et al. found that GWG below IOM guidelines reduced LGA and macrosomia risk without increasing SGA risk, suggesting lower GWG may benefit GDM women (39). Kurtzhals L conducted a cluster randomized controlled trial involving 44 medical institutions and 2,014 women, customizing personalized end-of-pregnancy weight targets based on pre-pregnancy BMI and GWG trajectories at the time of GDM diagnosis. Although there were no statistically significant differences in pregnancy outcomes between the intervention group and the routine care group, the incidence of LGA was significantly lower in the intervention group without an increase in the risk of SGA, suggesting that weight gain below the recommended level may be beneficial for women with GDM (40). Wong T’s study indicated that pregnant women with GDM who gained more weight than the IOM standards had an increased risk of cesarean section, LGA, and macrosomia, while the risk of SGA decreased (37). Contrary to the studies mentioned above, this study shows that in the normal weight group, GWG below the recommended range based on the IOM, CNS, or AOR standards increased the risk of SGA. Conversely, in the overweight group, GWG below the recommended range did not increase the risk of SGA. This suggests that in the normal weight population, maintaining GWG within the normal range helps reduce the occurrence of SGA, while in the overweight population, limiting excessive GWG is more meaningful.

After a GDM diagnosis, pregnant women typically control their diet and increase physical activity, slowing weight gain. GDM management in China follows a one-day outpatient model. GWG in GDM pregnancies can be divided into pre- and post-diagnosis stages. Studies have shown that the weight gain of pregnant women before being diagnosed with GDM is usually higher than that of non-GDM pregnant women (41). However, after diagnosis, their weight gain is lower than that of non-GDM pregnant women, resulting in the overall weight gain of GDM pregnant women being lower than that of non-GDM pregnant women (42). Therefore, in the management of GDM, weight monitoring and regular ultrasound examinations of fetal growth and development are critical, in addition to blood glucose monitoring. Although GWG is a relatively easy-to-obtain indicator, it is actually a composite outcome reflecting the combined effects of maternal fat deposition, pregnancy-related plasma volume expansion, hypertrophy of mammary and uterine tissues, extracellular fluid, placental mass, fetal mass, and amniotic fluid volume (43). These findings emphasize the complexity of managing GWG in pregnant women with GDM. More rigorous research designs and consideration of multiple influencing factors are needed to determine the ideal weight gain range.

This study has several advantages. Firstly, it is based on data from a large number of singleton pregnancies with GDM, examining the relationship between GWG and perinatal outcomes, and attempting to determine the optimal GWG range. Secondly, the study provides personalized pregnancy guidance for women with different pre-pregnancy weights by setting GWG targets based on BMI categories. However, the study also has limitations. Firstly, relying on self-reported pre-pregnancy weight may introduce recall bias. Secondly, due to the lack of weight data at the time of GDM diagnosis in the retrospective database, and the inability to include relevant indicators such as total dietary energy intake and blood glucose control (e.g., glycated hemoglobin) in the adjustment model, future research needs to be further designed and validated in a prospective singleton pregnancy cohort. Finally, limited by sample size, this study could not provide more precise and detailed estimates for the appropriate GWG range for underweight and obese pregnant women, and it did not conduct comprehensive stratification in analyzing the relationship between GWG and specific adverse outcomes.

## Conclusion

GWGis closely associated with adverse pregnancy outcomes. For singleton pregnant women with GDM who have a normal pre-pregnancy BMI, the GWG ranges recommended by the CNS and AOR standards are more suitable for Chinese pregnant women.

## Data Availability

The raw data supporting the conclusions of this article will be made available by the authors, without undue reservation.
